# Identification of Novel Biomarkers for Sepsis Prognosis via Urinary Proteomic Analysis Using iTRAQ Labeling and 2D-LC-MS/MS

**DOI:** 10.1371/journal.pone.0054237

**Published:** 2013-01-23

**Authors:** Longxiang Su, Lichao Cao, Ruo Zhou, Zhaoxu Jiang, Kun Xiao, Weijing Kong, Huijuan Wang, Jie Deng, Bo Wen, Fengji Tan, Yong Zhang, Lixin Xie

**Affiliations:** 1 Department of Respiratory Medicine, Hainan Branch of Chinese PLA General Hospital, Sanya, Hainan Province, China; 2 Medical College, Nankai University, Tianjin, China; 3 Shenzhen Proteome Engineering Laboratory, BGI Shenzhen, Shenzhen, China; 4 Department of Pediatrics, First Hospital, Peking University, Beijing, China; 5 Department of Respiratory Medicine, Chinese PLA General Hospital, Beijing, China; Moffitt Cancer Center, United States of America

## Abstract

**Objectives:**

Sepsis is the major cause of death for critically ill patients. Recent progress in proteomics permits a thorough characterization of the mechanisms associated with critical illness. The purpose of this study was to screen potential biomarkers for early prognostic assessment of patients with sepsis.

**Methods:**

For the discovery stage, 30 sepsis patients with different prognoses were selected. Urinary proteins were identified using isobaric tags for relative and absolute quantitation (iTRAQ) coupled with LC-MS/MS. Mass spec instrument analysis were performed with Mascot software and the International Protein Index (IPI); bioinformatic analyses were used by the algorithm of set and the Gene Ontology (GO) Database. For the verification stage, the study involved another 54 sepsis-hospitalized patients, with equal numbers of patients in survivor and non-survivor groups based on 28-day survival. Differentially expressed proteins were verified by Western Blot.

**Results:**

A total of 232 unique proteins were identified. Proteins that were differentially expressed were further analyzed based on the pathophysiology of sepsis and biomathematics. For sepsis prognosis, five proteins were significantly up-regulated: selenium binding protein-1, heparan sulfate proteoglycan-2, alpha-1-B glycoprotein, haptoglobin, and lipocalin; two proteins were significantly down-regulated: lysosome-associated membrane proteins-1 and dipeptidyl peptidase-4. Based on gene ontology clustering, these proteins were associated with the biological processes of lipid homeostasis, cartilage development, iron ion transport, and certain metabolic processes. Urinary LAMP-1 was down-regulated, consistent with the Western Blot validation.

**Conclusion:**

This study provides the proteomic analysis of urine to identify prognostic biomarkers of sepsis. The seven identified proteins provide insight into the mechanism of sepsis. Low urinary LAMP-1 levels may be useful for early prognostic assessment of sepsis.

**Trial Registration:**

ClinicalTrial.gov NCT01493492

## Introduction

Sepsis is a challenge to the field of critical care medicine. In the United States alone, 751,000 new patients suffer from severe sepsis annually, resulting in 210,000 deaths [1]. Sepsis has diverse, non-specific clinical manifestations, complicating its diagnosis and the assessment of its severity and prognosis [2]. If sepsis is not controlled in time, lasting and excessive inflammation can stimulate the progression of moderate sepsis to severe sepsis or even multiple organ dysfunction (MOD) [3]. Sepsis-associated MOD is one of the main causes of death of intensive care unit (ICU) patients. Therefore, the prognosis of sepsis must be assessed as early as possible.

The kidney is one of the organs most easily damaged by sepsis [4]. When sepsis occurs, hemodynamic factors may play a role in the loss of GFR. Non-hemodynamic mechanisms of cell injury, such as inflammatory, immunologic, and toxic events, are likely to cause kidney dysfunction [5]. Therefore, molecules present in urine have the potential for use as diagnostic biomarkers. Such biomarkers would be particularly suitable for sepsis because they could be obtained easily and non-invasively, facilitating close surveillance and reducing the cost of medical care. In general, urine is an ideal source of biomarkers because urine contains large numbers of small peptides [6].

Recently, proteomic technologies have been used to search for new biomarkers. A large number of differentially expressed proteins have been identified and reported as potential biomarkers for the diagnosis and prognosis of several different diseases. High-throughput urinary proteomics has been widely utilized to identify potential candidates for diagnostic and prognostic biomarkers and new therapeutic targets in renal research [7]. The isobaric tags for relative and absolute quantitation (iTRAQ) technique is one of the most widely used approaches. It can simultaneously analyze 4–8 different specimens, thus increasing throughput while reducing experimental error [8]. iTRAQ labeling coupled with LC-MS/MS is sensitive, automated, and multidimensional and can detect large molecules (>20 kDa) [9]. Especially, ProteoMiner technology can be used for helping iTRAQ quantitative analysis of low abundant proteins in complex biological samples [10]. iTRAQ is suitable for exploratory studies of the pathogenic mechanisms and pathophysiology of diseases [11]. In this study, we applied iTRAQ combined with two-dimensional liquid chromatography-tandem mass spectrometry (2D-LC-MS/MS) to investigate protein profiles in sepsis patients with different prognoses to identify potential prognostic biomarkers.

## Materials and Methods

### Recruitment of Subjects

Subjects were selected from inpatients who were hospitalized between May 2010 and Oct 2011 in the Respiratory ICU, Surgical ICU, and Emergency ICU at Chinese People's Liberation Army (PLA) General Hospital. Sepsis was diagnosed according to the 1991 ACCP/SCCM Sepsis Directory [12] and the diagnosis criteria advanced by the 2001 and 2008 International Sepsis Definition Conference [13], [14]. Patients exhibiting two or more of the following signs were eligible for Systemic Inflammatory Response Syndrome (SIRS) diagnosis: (1) temperature of >38°C or <36°C, (2) pulse rate of >90 beats/min, (3) respiratory rate of >20 breaths/min or hyperventilation with a partial pressure of arterial carbon dioxide (PaCO2) of <32 mmHg, or (4) white blood cell (WBC) count of >12,000 µL^−1^ or <4000 µL^−1^, or >10% immature cells. The presence of infection, defined according to the clinical and microbiological criteria of the CDC definitions [15]. Sepsis is a potentially deadly medical condition characterized by SIRS that is triggered by an clear infection. Finally, thirty patients diagnosed with sepsis were included in the discovery stage using iTRAQ. According to their different prognoses, these sepsis patients were further divided into a survivor group (≥28-day survival) and a matched-pairs non-survivor group (died during treatment). Urine specimens from survivors were collected from sepsis patients within 24 h of admission to the ICU (15 cases); urine specimens of non-survivors were collected from sepsis patients within 48 h before death (15 cases). For the control group, an additional 15 SIRS patients but negative pathologic examination were selected from the SICU within 24 hours after aseptic surgery. As mentioned above, samples were classified into the SIRS control group (SI), the survivors-sepsis group (SP), and the non-survivors-sepsis group (de). Another 54 patients were included in the verification stage. Again, patients were pair-matched and evenly classified into survivor (≥28 days) and non-survivor groups (<28 days) according to 28-day survival. However, urine samples of verification-stage patients were all collected within 24 hours after ICU admission to screen for early biomarkers of prognostic assessment. Patients were excluded if they were younger than 18 years of age; suffered from anuria or urinary tract infections; contracted acquired immunodeficiency syndrome; had reduced polymorphonuclear granulocyte counts (<500 µL^−1^); were receiving dialysis treatment for chronic kidney disease; died within 24 h after admission to the ICU; refused to participate in the study; or declined treatment during the period of observation. Patients or their family members were fully informed of the study and signed informed consent forms of their own accord. This study was approved by the Ethics Committee of the CPLA General Hospital (project No. 20111013-007) and was registered with the U.S. National Institutes of Health Clinical Trials Register (NCT01493492).

### Reagents and Chemicals

Reagent grade chemicals were purchased from Sigma Aldrich (Oakville, ON, Canada) or Fisher Scientific (Nepean, ON, Canada). All iTRAQ reagents and buffers were obtained from Applied Biosystems Inc. (ABI, Foster City, CA).

### Urine Sample Preparation

All urine samples were collected through Foley catheters in the morning. For the discovery stage, urine specimens from sepsis survivors (15 cases) and SIRS controls (15 cases) were collected from these patients within 24 h of admission to the ICU; urine specimens of non-survivors were collected from sepsis patients within 48 h before death (15 cases). It's worth nothing that urine samples of verification-stage were different from discovery stage. That is to say, patients' urine sample involved in verification-stage were collected within 24 hours after ICU admission. Each urine sample was centrifuged at 2,000 rpm for 10 minutes at 4°C. The supernatants were transferred into Eppendorf tubes and stored at −80°C.

Sample pooling is a commonly used strategy to reduce the influence of individual variation on candidate target selection in proteomic studies [16]. For the iTRAQ, urine proteins from patients with the same stages and the controls were pooled into a subgroup to minimize individual variation and enhance signals. Three independent sample subgroups, SI, SP, and de, were used for biomarker discovery by iTRAQ labeling. Another 54 patients classified into survivor and non-survivor groups and were assessed with Western blots.

### Concentration, Desalting, and Quantitation of Urine Samples

Individual urine samples were centrifuged at 8000× g for 10 min at 4°C before experiment. The supernatants of samples from patients with the same histological grades or pathological stages were mixed as a subgroup. Each subgroup had a volume of at least 50 mL. The mixtures were then enriched and desalted with a 1-kDa centrifugal filter as described by the manufacturer (Spectrum, CA, USA); this process reduced the volume per subgroup to approximately 500 µl. To reduce the protein concentration range of the samples, a ProteoMiner™ Protein Enrichment Large-Capacity Kit (BIO-RAD, 163-3007) was used according to the manufacturer's instructions. After elution, the protein content of each subgroup was quantified with a Bradford Protein Assay Kit (CWBIO, CW0013).

### iTRAQ Labeling and Strong Cationic Exchange (SCX) Fractionation

One hundred mictoliters of the processed protein were taken out of each sample solution and then the protein was digested with Trypsin Gold (Promega, Madison, WI, USA) with the ratio of protein ∶ trypsin = 30 ∶ 1 at 37°C for 16 hours. After trypsin digestion, peptides were dried by vacuum centrifugation. Peptides were reconstituted in 0.5 M TEAB and processed according to the manufacturer's protocol for 8-plex iTRAQ (Applied Biosystems). Briefly, one unit of iTRAQ reagent (defined as the amount of reagent require to label 40 µg of protein) was thawed and reconstituted in 24 µL isopropanol. Peptides were labeled with 116, 118 and 121 iTRAQ tags by incubation at room temperature for 2 h. The labeled peptide mixtures were then pooled and dried by vacuum centrifugation.

SCX chromatography was performed with a Shimadzu LC-20AB HPLC Pump system. The iTRAQ-labeled peptide mixtures were reconstituted with 4 mL buffer A (25 mM NaH_2_PO_4_ in 25% ACN, pH 2.7) and loaded onto a 4.6×250 mm Ultremex SCX column containing 5-µm particles (Phenomenex). The peptides were eluted at a flow rate of 1 mL/min with a gradient of buffer A for 10 min, 5–35% buffer B (25 mM NaH_2_PO_4_, 1 M KCl in 25% ACN, pH 2.7) for 11 min, 35–80% buffer B for 1 min. The system was then maintained at 80% buffer B for 3 min before equilibrating with buffer A for 10 min prior to the next injection. Elution was monitored by measuring the absorbance at 214 nm, and fractions were collected every 1 min. The eluted peptides were pooled into 10 fractions, desalted with a Strata X C18 column (Phenomenex) and vacuum-dried.

### LC-ESI-MS/MS Analysis

Each fraction was resuspended in buffer A (2% ACN, 0.1% FA) and centrifuged at 20,000× *g* for 10 min. In each fraction, the final concentration of peptides was approximately 0.25 µg/µl. Using an autosampler, 20 µl of supernatant was loaded onto a 2 cm C18 trap column (inner diameter 200 µm) on a Shimadzu LC-20AD nanoHPLC. Peptides were eluted onto a resolving 10 cm analytical C18 column (inner diameter 75 µm) that was assembled in-house. The samples were loaded at 15 µl/min for 4 min and eluted with a 44-min gradient at 400 nl/min from 2 to 35% B (98% ACN, 0.1% FA), followed by a 2-min linear gradient to 80% B, maintenance at 80% B for 4 min, and finally a return to 2% B over 1 min.

**Figure 1:**
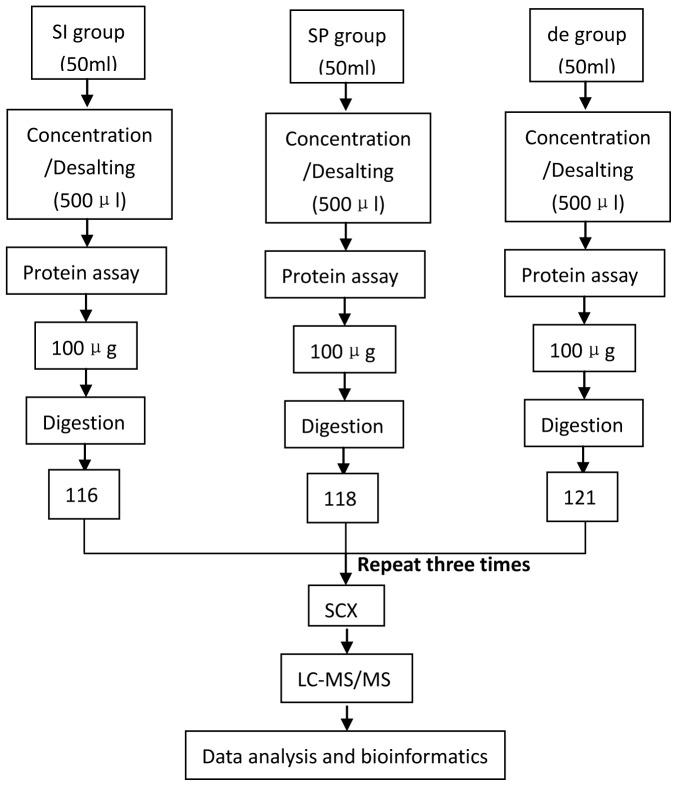
Schematic of the experimental design based on iTRAQ labeling combined with 2-D LC-MS/MS analysis of SI, SP, and de. SCX, strong cation exchange. SI: urine specimens from patients with SIRS. SP: urine specimens from sepsis patients, acquired within 24 h of admission to the ICU. de: urine specimens from sepsis patients, acquired within 48 h before death.

The peptides were subjected to nanoelectrospray ionization followed by tandem mass spectrometry (MS/MS) in an LTQ Orbitrap Velos (Thermo) coupled inline to the HPLC. Intact peptides were detected in the Orbitrap at a resolution of 60,000. Peptides were selected for MS/MS using the high-energy collision dissociation (HCD) operating mode with a normalized collision energy setting of 45%. Ion fragments were detected in the LTQ. A data-dependent procedure that alternated between one MS scan followed by eight MS/MS scans was applied for the eight most abundant precursor ions above a threshold ion count of 5,000 in the MS survey scan with the following Dynamic Exclusion settings: repeat counts, 2; repeat duration, 30 s; and exclusion duration, 120 s. The applied electrospray voltage was 1.5 kV. Automatic Gain Control (AGC) was used to prevent overfilling of the ion trap; 1×10^4^ ions were accumulated in the ion trap to generate HCD spectra. For MS scans, the *m/z* scan range was 350 to 2,000 Da. The experiment was repeated three times, and the results were categorized as SI, SP, and de. The experimental flowchart is shown in Figure 1.

### Database Search and Bioinformatics

The resulting MS/MS spectra were searched against the International Protein Index (IPI) human sequence databases (version 3.83, HUMAN, 93,289 sequences) with MASCOT software (Matrix Science, London, U.K.; version 2.2). For protein identification and quantification, a peptide mass tolerance of 10 ppm was allowed for intact peptide masses and 0.05 Da for fragmented ions. One missed cleavage was allowed in the trypsin digests; carbamidomethylation of cysteine was considered a fixed modification, and the conversion of N-terminal glutamine to pyro-glutamic acid and methionine oxidation were considered variable modifications. All identified peptides had an ion score above the Mascot peptide identity threshold, and a protein was considered identified if at least one such unique peptide match was apparent for the protein. For protein-abundance ratios measured using iTRAQ, we set a 1.5-fold change as the threshold and a two-tailed *p*-value<0.05 to identify significant changes. The logic algorithm for set operations was applied to further screen for differentially expressed proteins. Gene Ontology (GO) functional classifications were analyzed with Blast2GO software (http://www.blast2go.org/), and GO enrichment analysis was performed to identify GO terms that were significantly enriched in differentially expressed proteins.

### Western Blot Validation

Western blot analyses were performed to confirm the presence of differentially expressed proteins. After desalting and concentrating urine proteins, the amount of protein in each urine sample was measured using the bicinchoninic acid method. Urine proteins from individual samples were resolved on SDS PAGE gels and transferred electrophoretically onto PVDF membranes (Applygen Gene Technology Corp, Beijing, China). The membranes were blocked for 1 h at room temperature with 5% non-fat dried milk in Tris-buffered saline containing 0.1% Tween 20 (TBST, Applygen Gene Technology Corp). The following antibodies were used for Western blot analysis: anti-heparan sulfate proteoglycan 2 antibody (anti-HSPG2, 1∶1000, ab23418, Abcam, U.K.), anti-selenium binding protein 1 antibody (anti-SELENBP1, 1∶1000, sc-373726, Santa Cruz Biotechnology, Inc., CA, USA), and anti-lysosome-associated membrane proteins antibody (anti-LAMP1, 1∶1000, sc-17768, Santa Cruz Biotechnology, Inc.). The membranes were then washed with TBST three times and incubated with horseradish peroxidase-conjugated secondary antibody (Applygen Gene Technology Corp). Images were acquired by exposure to Kodak ×500 film (Midwest Scientific, Valley Park, MO).

### Statistical Analysis

Results for continuous variables with normal distributions are given as means ± standard deviations (SD). Student's t-test was used to compare means between two groups. The results for continuous variables that were not normally distributed are given as medians (interquartile range) and were compared with the Mann-Whitney U test. The results for qualitative variables were expressed as percentages and compared between groups with a Chi-square test. Statistical analyses were conducted with SPSS 16.0 (SPSS, Chicago, IL, USA), and a two-tailed *p*<0.05 was considered significant.

## Results

### Demographics of subjects

This study consists of two parts, the discovery stage and the verification stage. Table 1 lists the clinical information for all patients involved in this study. The discovery stage included 45 subjects, of which 15 were diagnosed with SIRS and 30 with sepsis with differing prognoses. The C-reactive protein (CRP) and Procalcitionin (PCT) levels of the two sepsis groups were higher than those of the SIRS group (*p* = 0.003 and *p* = 0.004, respectively), while there was no obvious difference between the survivor and non-survivor group. Acute Physiology, Age and Chronic Health E valuation II (APACHE II) scores decreased markedly from high to low in the following order: non-survivors>survivors>SIRS (*p*<0.001). Non-survivors had higher Sequential Organ Failure Assessment (SOFA) scores than survivors (*p*<0.001). Statistically, there were no differences in gender, age, WBC, or other etiological factors between these groups. For the verification stage, we selected another 54 subjects with sepsis who had been admitted to the ICU within 24 hours. These patients were further assigned to a survivor group (27 cases) and a non-survivor group (27 cases), according to 28-day survival. Of the clinical parameters, only APACHE II and SOFA scores were significantly different between survivors and non-survivors (*p*<0.001).

**Table 1:** Demographics of subjects in the discovery and verification stages.

Characteristics	SIRS	survivors	non-survivors	P value	survivors	non-survivors	P value
	N = 15	N = 15	N-15		N = 27	N = 27	
**Age** (years)	52±15	63±19	62±16	0.088	54±21	60±15	0.213
**Gender** (n, %)				0.228			0.761
Male	6(40)	10(66)	9(60)		19(70)	20(74)	
Female	9(60)	5(33)	6(40)		8(29.6)	7(26)	
**WBC counts**(×10^∧^9/L)	12.1±2.3	13.1±7.0	14.5±7.7	0.446	13.4±5.2	12.3±6.2	0.487
**Serum CRP** (mg/dl)	5.5±5.8	12.9±7.0	11.2±6.7	0.003	10.3±7.4	11.2±10.6	0.742
**Serum PCT** (ng/ml)	0.3(0.6)	3.0(7.1)	4.9(9.4)	0.004	1.2(5.7)	6.0(22.8)	0.169
**APACHE II score**	8±7	14±4	16±8	<0.001	15±7	21±7	<0.001
**SOFA score**	-	8±4	12±4	<0.001	6±4	9±4	<0.001
**Pathogens detected** (n, %)							
Gram-positive bacteria	-	6(40)	7(47)	0.713	9(33)	8(30)	0.77
Gram-negative bacteria	-	14(93)	15(100)	0.309	25(93)	26(96)	0.552
Fungi	-	7(47)	8(53)	0.191	11(41)	13(48)	0.669
**Etiological factors** (n, %)							
Pulmonary infection	-	12(80)	14(93)	0.283	17(63)	20(74)	0.379
Abdominal infection	-	6(40)	5(33)	0.705	4(15)	5(19)	0.715
Bacteremia	-	3(20)	6(40)	0.232	3(11)	6(22)	0.273
Trauma/postoperative infection	-	7(47)	9(60)	0.464	7(26)	5(19)	0.513

Quantitative data of normal distribution are presented as mean±SD. Quantitative data of non-normal distribution are presented as median (interquartile range). Qualitative data are presented as n(%).

WBC counts, white blood cell counts; CRP, C-reactive protein; PCT, Procalcitionin; APACHE II score, Acute Physiologic Assessment and Chronic Health Evaluation II scores; SOFA score, Sequential Organ Failure Assessment scores.

### Global description of urinary proteomic characteristics and reproducibility

**Figure 2:**
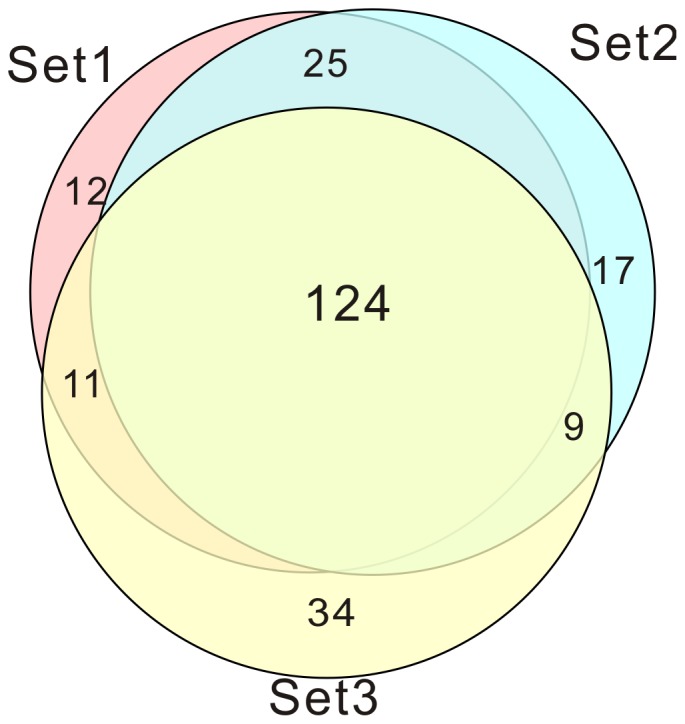
Proteins identified in the three sets (technical replicates).

Three technical replicates were analyzed to demonstrate the reproducibility of the experimental results and to perform complementation. Figure 2 compares the proteins identified in the three sets (technical replicates). A total of 232 proteins were identified, of which 124 proteins were found in all three sets. More specifically, 172 unique proteins (501 distinct peptides), 175 unique proteins (510 distinct peptides) and 178 unique proteins (528 distinct peptides) were identified in set 1, set 2 and set 3, respectively. Of these, 147 (73.5%) were identified in both sample set 1 and set 2, 134 (62%) were in both set 1 and set 3, and 133 (60.5%) were in both set 2 and set 3. According to our criteria for judging differentially expressed proteins (fold change ratio≥1.5 and *p*-value<0.05), 29, 32 and 45 up-regulated proteins were identified in the SP/SI, de/SI and de/SP comparisons, respectively. Similarly, 32, 11 and 23 down-regulated proteins were identified (Table 2).

**Table 2:** Summary of differentially expressed proteins identified in SP/SI, de/SI and de/SP among the three sets.

	SP/SI		de/SI		de/SP	
	up	down	up	down	up	down
**set1**	31	30	26	11	40	27
**set2**	28	30	34	8	47	18
**set3**	29	38	36	15	48	23
**average**	29	32	32	11	45	23

SI: urine specimens from patients with SIRS.

SP: urine specimens from sepsis patients, acquired within 24 h of admission to the ICU.

de: urine specimens from sepsis patients, acquired within 48 h before death.

de/SI: proteins differentially expressed in de relative to SI.

de/SP: proteins differentially expressed in de relative to SP.

SP/SI: proteins differentially expressed in SP relative to SI.

### Bioinformatics

**Figure 3:**
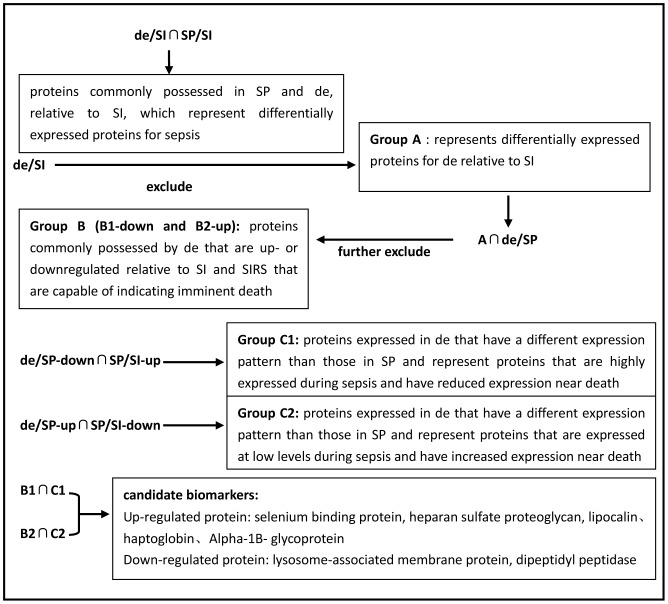
The analytical process for candidate target protein identification. SI: urine specimens from patients with SIRS. SP: urine specimens from sepsis patients, acquired within 24 h of admission to the ICU. de: urine specimens from sepsis patients, acquired within 48 h before death. de/SI: proteins differentially expressed in de relative to SI. de/SP: proteins differentially expressed in de relative to SP. SP/SI: proteins differentially expressed in SP relative to SI.

To identify candidate sepsis prognostic biomarkers, we analyzed the experimental results based on biomathematics as follows (Figure 3): (1) The initial dataset contained all proteins identified as differentially expressed at least twice in the experiment. (2) de is sepsis at the end stage. To exclude proteins which do not include this stage, we first filter out proteins reflecting sepsis. Proteins that were differentially expressed in sepsis cases (SP/SI ∩ de/SI) were excluded from de/SI; the resultant proteins were named Group A, which represents differentially expressed proteins of de with respect to SI. (3) Group A was overlapped with de/SP. The proteins obtained (Group A proteins that overlapped with de/SP) were named Group B, which represents a bad prognosis, and were sub-classified into up-regulated proteins and down-regulated proteins, named B1 and B2, respectively. (4) From another perspective, as sepsis worsened, the body's immune system weakened, resulting in the down-regulation of some proteins prior to the patient's death, although these proteins were expressed relatively higher during the sepsis period, or vice versa. Hence, the proteins with opposite trends were overlapped in pairs, and these proteins were named C1 and C2: C1 includes the proteins that were highly expressed during the sepsis period but were decreased prior to death, whereas C2 includes the opposite cases. (5) We then selected differentially expressed proteins that were informative regarding prognosis, i.e., proteins that showed the same trend in expression in both Group B and Group C. The resulting group of proteins was associated with poor prognosis. Using these criteria, seven proteins were identified as being differentially expressed in sepsis cases but showed dissimilar trends regarding prognosis. Of these proteins, five (SELENBP-1, HSPG-2, A-1-BG, HPR, LCN) were up-regulated in de and down-regulated in SP, and two (LAMP-1, DPP-4) were down-regulated in de and up-regulated in SP. MS/MS spectra for these potential biomarkers for sepsis prognosis have been uploaded to Peptideatlas database. The raw MS data is publicly available for download from PeptideAtlas now (available at: http://www.peptideatlas.org/PASS/PASS00105).

**Figure 4:**
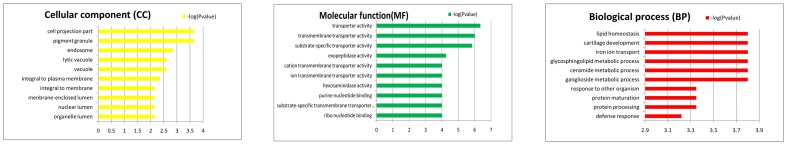
GO annotation of the final selected differentially expressed proteins. These differentially expressed proteins were classified among three categories: cellular component (CC), molecular function (MF) and biological process (BP). According to the GO database, the top 10 components for CC, MF, BP of the selected differentially expressed proteins are shown along with their enrichment score, represented as a *p*-value.

The final selected differentially expressed proteins were analyzed using the GO database to determine their cellular-component association, molecular function, and participation in biological processes (Figure 4). In the cellular component analysis, we found that most of the potential biomarkers were associated with the cell projection part, pigment granule, endosome, vacuole, membrane or lumen regions. In the molecular function analysis, most of these proteins were found to play a role in transport, enzymatic activity, purine nucleotide binding or ribonucleotide binding. In the biological process analysis, these proteins were mainly involved in lipid homeostasis, cartilage development, iron ion transport, or select metabolic processes. These differentially expressed proteins may be related to several metabolic pathways, including phagosome and lysosome, protein digestion and absorption, arachidonic acid metabolism, ECM-receptor interaction, metabolic pathways, complement and coagulation cascades.

### Western Blot Validation

**Figure 5:**
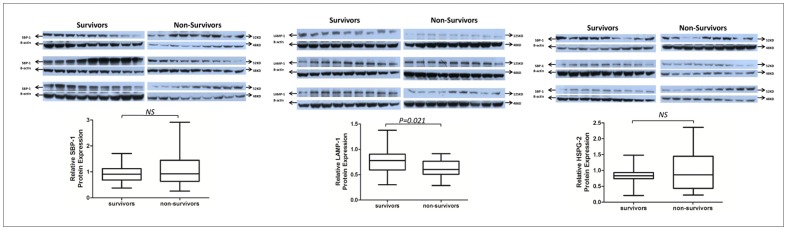
Western blot validation of three candidate markers in individual sepsis patients with different prognoses. (A) Relative protein expression of SBP-1. The survivor group and non-survivor groups were 0.938±0.347 and 0.945±0.602 (*p*>0.05), respectively. (B) Relative protein expression of LAMP-1. The survivor group and non-survivor groups were 0.752±0.246 and 0.617±0.166 (*p*<0.05), respectively. (C) Relative protein expression of HSPG-2. The survivor and non-survivor groups were 0.802±0.282 and 0.880±0.606 (*p*>0.05), respectively.

Three of the identified differentially expressed proteins were validated by Western immunoblotting (Figure 5). LAMP-1 protein expression was significantly decreased in non-survivors (*p*<0.05). However, there was no difference in the expression of SBP-1 and HSPG-2 between the survivor and non-survivor groups (*p*>0.05).

## Discussion

This study used iTRAQ labeling followed by 2D-LC-MS/MS for the quantitative proteomic analysis of pooled urine samples from sepsis patients with different prognoses and from SIRS controls to discover candidate biomarkers for sepsis prognosis. Using bioinformatics, we identified seven differentially expressed proteins: LAMP-1, SELENBP-1, HSPG-2, A-1-BG, HPR, LCN, and DPP-4. Previous studies have reported associations between four of these proteins and sepsis or related diseases. Therefore, we examined the remaining three candidate proteins, which have not been extensively studied. The expression of LAMP-1 in the early stage of sepsis was significantly lower in non-survivors, suggesting that LAMP-1 is a biomarker for early prognosis assessment.

Our mass spectrometry results indicate that HPR, A-1-BG, and LCN-1 are proteins that are up-regulated with a bad prognosis. Haptoglobin (HPR) expression is known to be increased in inflammation, infection, and cancer [17]. Using surface-enhanced laser desorption/ionization time-of-flight mass spectrometry (SELDI-TOF-MS), Vanhoutte et al. found an increase in haptoglobin in the urine of patients with early renal injury [18]. Kalenka et al. also identified haptoglobin in the serum proteomes of patients with sepsis and septic shock [19]. α-1-B-Glycoprotein (A-1-BG) is a member of the immunoglobulin superfamily and a known plasma protein. By 2D-MS, Kalenka et al. demonstrated that A-1-BG was more highly up-regulated in non-survivors than in sepsis survivors [19]. Piyaphanee et al. first validated α-1-B glycoprotein fragmentation as a urinary biomarker in pediatric steroid-resistant nephrotic syndrome [20]. Neutrophil gelatinase-associated lipocalin/lipocalin-2 has emerged as an excellent stand-alone troponin-like biomarker in urine and plasma for the prediction, monitoring of clinical trials, and prognosis of AKI [21]. Maddens et al. identified urinary biomarkers for septic AKI via shotgun proteomics in a mouse model of sepsis and validated these biomarkers in individual urine samples of mice and human sepsis patients with and without AKI. They found that urinary NGAL was the most distinctive biomarker for septic AKI [22]. In addition, dipeptidyl peptidase-4 (DPP-4) expression in non-survivors was significantly lower than in survivors. Bergmann et al. measured DPP-4 concentrations with a fluorometric assay and found that sepsis patients had decreased DPP-4 activity compared to control individuals. Bergmann et al. correlated the significant decrease in DPP-4 activity in patients with severe sepsis with an increase in procalcitonin [23]. These observations corroborate our mass spectrometry results.

No relationship between LAMP-1, SELENBP-1, or HSPG-2 has been previously reported. To verify the existence of these proteins by Western blotting, we selected another matched-pair sepsis cohort of 54 patients with different prognoses based on 28-day survival. Urine specimens were collected from these patients within 24 hours of ICU admission to identify a sensitive, early biomarker for sepsis prognosis. WB results are shown in Figure 5 Only LAMP-1 decreased significantly in the non-survivor group at the early stage (*p*<0.05). This discrepancy with the mass spectrometry data may be due to the difference in the sample collection time in the discovery- and validation-stage patients. Although the difference in SELENBP-1 and HSPG-2 expression in the two groups was not statistically significant, their expression was consistent with expectations, i.e., the expression of SELENBP-1 and HSPG-2 in the non-survivor group was higher than that in the survivor group at this earlier stage; expression levels may continue to rise as the disease progresses.

Lysosome-associated membrane proteins have proven to be useful immunological and biochemical markers of lysosomes, which are membrane-bound organelles that play an important role in antigen presentation, cholesterol metabolism, and hydrolysis of exogenous materials that enter the cell by endocytosis, pinocytosis, or phagocytosis [24], [25]. LAMPs likely contribute to the migration of activated leukocytes to sites of inflammation *in vivo* and mediate inflammation and the immune response of patients with auto-immune diseases [26]. Recently, studies have shown that CD107a (LAMP-1) may be a marker for the de-granulation of NK, activated CD8+ T cells and activation-induced secretion of human mast cells [27]–[29]. These findings illustrate the importance of the LAMP-1-mediated immune and inflammatory response. Künzli et al. observed that LAMP-1 expression favors local tumor progression rather than tumor metastasis in pancreatic carcinoma. Their study revealed that patients with tumors with high LAMP-1 mRNA expression lived significantly longer after tumor resection than patients with tumors with low to moderate LAMP-1 mRNA levels [30]. This correlation demonstrates that LAMP-1 can be used as a prognostic marker. In our study, LAMP-1 expression levels in non-survivors are significantly lower than those in survivors. This difference may be due to impairment or lack of immune function in patients with poor prognosis. However, the specific mechanism is unclear and remains to be explored.

Selenium binding protein (SBP) exerts a chemoprotective effect via several mechanisms [31], [32], including inhibition of cell proliferation, cell cycle arrest, and promotion of cell death. SBP-1 expression is significantly lower or even undetectable in a variety of epithelial tumor tissues, including lung [33], colorectal [34], and ovarian cancers [35]. Xia et al. used proteomic analysis to identify specific disease-associated proteins as potential clinical biomarkers in gastric cancer and found SBP-1 to be differently expressed. Their study demonstrated that the 3-year survival rate of patients with high SBP-1 expression was significantly higher than that of patients with low expression. They concluded that SBP-1 is a biomarker for gastric cancer prognosis [36]. Zhang et al. [37] and Li et al. [34] have also demonstrated a relationship between SBPs and the prognosis of gastrointestinal carcinoma. Differences in the expression of SBPs in animals with ischemia/reperfusion lung injury or severe burns and *Pseudomonas aeruginosa* sepsis have also been identified in proteomic studies [38], [39]. Heparan sulfate proteoglycan binds to and regulates many inflammatory mediators *in vitro* [40], [41], suggesting that it serves an important role in directing the progression and outcome of inflammatory responses *in vivo*. A study evaluating glycocalix changes in patients with sepsis compared patients after major abdominal surgery and healthy volunteers. Levels of HSGP-1 were higher in the sepsis group compared with the control group and lower than in the surgery abdominal group [42]. Oragui et al. observed that urinary excretion of heparan sulfates was significantly increased in meningococcal disease [43]. As demonstrated by Western blotting, although the two types of proteins, SBP-1 and HSGP-1, were more highly expressed in the non-survivor group than in the survivor group, the difference was not statistically significant. Therefore, these two proteins are unsuitable for use as biomarkers for prognosis in early stage sepsis. However, as the disease progresses, these two proteins became more highly expressed in sepsis patients who died. These proteins may therefore aid in the study of sepsis pathogenesis.

The present study has some limitations. First, the kidney is one of the most easily damaged organs during the sepsis process. Therefore, at the initial stage of sepsis, changes will immediately occur in the kidney. However, these small but very important changes have not yet been included in our clinical diagnostic criteria for impaired renal function. Although we excluded patients suffering from anuria and urinary tract infection or undergoing dialysis treatment for chronic kidney disease, our study does not exclude patients with acute kidney injury. Sepsis patients with acute kidney injury require further study. Another limitation is that the etiology and clinical manifestations of ICU inpatients are extremely complex. The more serious the condition, the more complex the cause. Patients with a wide range of clinical presentations were inevitably involved in the study. Though we analyzed pooled clinical samples to reduce the influence of individual variation on biomarker selection, the sample size of discovery stage is limited and we did not exclude the influence of sepsis heterogeneous of these patients enrolled. This study is preliminary, so future studies are necessary to examine the effects of different sepsis etiologies, levels of organ damage, and sepsis severity. Finally, the sample size was not sufficiently large to demonstrate that low levels of LAMP-1 indicate poor prognosis, and SBP-1 and HSPG-2 with negative results. However, a power analysis was used to calculate by R language how many samples would be fitting here if there was no way of obtaining more samples for validation. We found that 27 samples in each group is able to meet the minimum sample size in the validation stage (see Table S1). Additionally, because of the limitations of Western blotting, we could not perform quantitative measurements of LAMP-1. Larger sample sizes and a more quantitative study are necessary to validate our conclusions.

## Conclusions

This study provides the analysis of urine samples using iTRAQ to measure relative protein expression in sepsis patients to identify prognostic candidate biomarkers. We demonstrated that this screening method has potential valuable in studying sepsis. We identified a number of differentially expressed proteins that may be associated with sepsis prognosis. However, our study is to illustrate the problem from a clinical point of view. In the future study, we want to prove the conclusion through clinical specimens of the large sample size.

## Supporting Information

Table S1 Sample size estimation for the verification stage used two-sample t test power calculation by R language.
(DOC)
