# Supplementary material for: Identification of Novel Biomarkers for Sepsis Prognosis via Urinary Proteomic Analysis Using iTRAQ Labeling and 2D-LC-MS/MS
Source: PLoS One. 2013 Jan 23;8(1):e54237. doi: 10.1371/journal.pone.0054237 (PMC3553154; doi:10.1371/journal.pone.0054237)
Supplement: Table S1 — Sample size estimation for the verification stage used two-sample t test power calculation by R language. (DOC) [file pone.0054237.s001.doc]

**Table S1 Sample size estimation for the verification stage used two-sample t test power calculation by R language**

| **Proteins** | **Groups** | **Delta** | **SD** | **Sample N** |
| --- | --- | --- | --- | --- |
| **LAMP-1** | survivors | 0.28 | 0.24 | 12.56 |
|  | non-survivors | 0.19 | 0.16 | 12.17 |
| **SBP-1** | survivors | 0.4 | 0.34 | 12.37 |
|  | non-survivors | 0.65 | 0.59 | 13.96 |
| **HSPG-2** | survivors | 0.3 | 0.28 | 14.69 |
|  | non-survivors | 0.69 | 0.6 | 12.9 |

significance level and power value were set as 0.05 and 0.8 respectively.

delta represents true difference in means; sd represents standard deviation; sample N represents estimated sample content.
